# Positioning *Bacillus subtilis* as terpenoid cell factory

**DOI:** 10.1111/jam.14904

**Published:** 2020-11-20

**Authors:** H. Pramastya, Y. Song, E.Y. Elfahmi, S. Sukrasno, W.J. Quax

**Affiliations:** ^1^ University of Groningen Groningen The Netherlands; ^2^ Pharmaceutical Biology Research Group School of Pharmacy Institut Teknologi Bandung Bandung Indonesia

**Keywords:** *Bacillus subtilis*, cell factory, MEP pathway, metabolic engineering, terpenoids

## Abstract

Increasing demands for bioactive compounds have motivated researchers to employ micro‐organisms to produce complex natural products. Currently, *Bacillus subtilis* has been attracting lots of attention to be developed into terpenoids cell factories due to its generally recognized safe status and high isoprene precursor biosynthesis capacity by endogenous methylerythritol phosphate (MEP) pathway. In this review, we describe the up‐to‐date knowledge of each enzyme in MEP pathway and the subsequent steps of isomerization and condensation of C5 isoprene precursors. In addition, several representative terpene synthases expressed in *B. subtilis* and the engineering steps to improve corresponding terpenoids production are systematically discussed. Furthermore, the current available genetic tools are mentioned as along with promising strategies to improve terpenoids in *B. subtilis*, hoping to inspire future directions in metabolic engineering of *B. subtilis* for further terpenoid cell factory development.

## Introduction

### The nature of *Bacillus subtilis*

*Bacillus subtilis*, a well‐known Gram‐positive bacterium, was one of the first organisms to have its genome successfully annotated. In academia, *B. subtilis* strain 168 has become a model micro‐organism for the study of physiological properties covering the proteome, protein secretory and translocation mechanisms, the cell division mechanism and last but not least the development of minimal cell bacteria. For the industries, *B. subtilis* 168 has been well‐known for its generally recognized as safe status facilitating easier purification of the protein or metabolites in the absence of endotoxin (Schallmey *et al*.^2004^).

Residing as a special niche of the soil microbial ecosystem, *B. subtilis* has its strength in metabolite production required for survival (Yang *et al*.^2016^). It is known that the bacterium has its capability to produce diverse secondary metabolites including polyketides and terpenoids acting as antimicrobial agents or being part of a defence mechanism against particular stresses (Calderone *et al*.^2006^; Butcher *et al*.^2007^; Bosak *et al*.^2008^; Kontnik *et al*.^2008^; Lee and Kim 2011; Barbosa *et al*.^2015^; Caulier *et al*.^2019^). However, the engineering of *B. subtilis* for metabolite production is lagging behind compared to *Escherichia coli* or *Saccharomyces cerevisiae* (Gu *et al*.^2018^). Numerous small organic molecules nonnative to these microbial hosts have been produced and many of them have reached the market (Schempp *et al*.^2018^). The reasons include the late development of diverse molecular tools and genome scale exploratory research that are required to facilitate precise engineering of the bacterium. Only during the past 10 years that more attention has been given to provide more tools for molecular engineering of the bacterium (Vavrová *et al*.^2010^; Wang *et al*.^2012^; Guiziou *et al*.^2016^; Popp *et al*.^2017^; Castillo‐Hair *et al*.^2019^). To give better perspective on *B. subtilis*, comparison among these three microbial platforms are available in Table 1.

**Table 1:** Comparison of *Escherichia coli*, *Saccharomyces cerevisiae* and *Bacillus subtilis* as metabolite cell factories

Microbial platform	Advantage	Disadvantage	References
*Escherichia coli*	Diverse and sophisticated molecular engineering tools Fast and easy to grow; has been a routine microbial platform in synthetic biology	Safety concern related to its endotoxin production nature Lack of endomembrane system for expression of eukaryotic CYP450 involving downstream steps of some terpenoid biosynthetic pathways	Dietrich *et al*.(2009); Zhou *et al*.(2013); Yang *et al*.(2020)
*Saccharomyces cerevisiae*	GRAS micro‐organism Diverse molecular engineering tools Possesses endomembrane system readily for CYP450 expression	Relatively slow growth More complex structure of the genome for engineering More difficult to put the whole heterologous pathway into the micro‐organism since limited capability in polycistronic expression	Zhou *et al*.(2013); Rahmat and Kang (2020); Yang *et al*.(2020)
*Bacillus subtilis*	GRAS bacterium Bacterium with considerably high isoprene emission Possessing potential CYP450s that can be developed for terpenoid oxidation, such as CYP109B1, CYP102A2 and CYP102A3. CYP109B1 has the ability to oxidize valencene (a sesquiterpene) to nootkatone Possesses potential glycosyltransferases that might be utilized for production of glycoside terpenoids. UDP‐glycosyltransferase (Yji) of *B. subtilis* was able to transfer glycosyl moiety to protopanaxadiol leading to unnatural ginsenoside	Limited molecular engineering tools especially for dynamic range of protein expression and genomic engineering. Nevertheless, more tools are currently investigated	Kuzma *et al*.(1995); Gustafsson *et al*.(2004); Schallmey *et al*.(2004); Girhard *et al*.(2010); Liang *et al*.(2017); Popp *et al*.(2017)

This review deals with the progress on engineering of *B. subtilis* as the microbial cell factory. Data on the basic knowledge of the biosynthesis pathway, especially related to bacteria or in particular *B. subtilis* are presented. Future perspectives based on the progress in synthetic biology and current cutting‐edge technology are also the focus of this review.

### *B. subtilis* terpenoids producing ability

**Figure 1:**
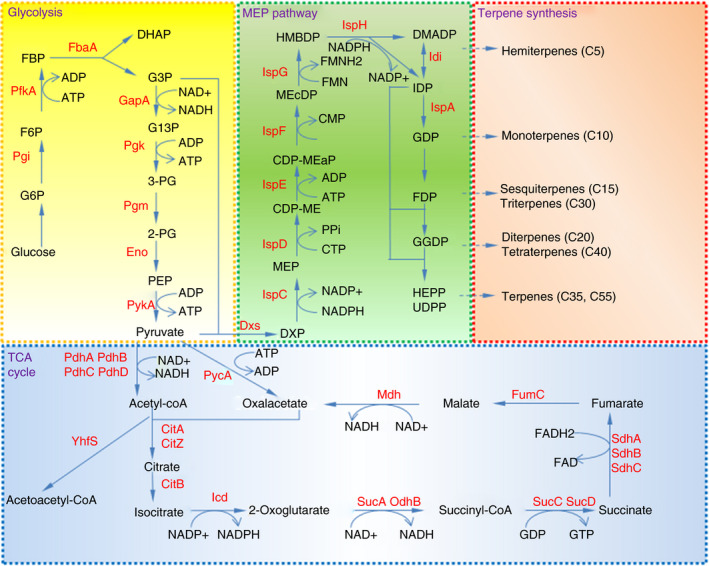
Scheme of MEP pathway, glycolysis and TCA cycle in *Bacillus subtilis* 168. Pgi, glucose 6‐phosphate isomerase; PfkA, phosphofructokinase; FbaA, fructose 1,6‐bisphosphate aldolase; GapA, glyceraldehyde 3‐phosphate dehydrogenase; Pgk, phosphoglycerate kinase; Pgm, phosphoglycerate mutase; Eno, enolase; PykA, pyruvate kinase; PdhA, pyruvate dehydrogenase (E1 alpha subunit); PdhB, pyruvate dehydrogenase (E1 beta subunit); PdhC, pyruvate dehydrogenase (dihydrolipoamide acetyltransferase E2 subunit); PdhD, dihydrolipoamide dehydrogenase E3 subunit; PycA, pyruvate carboxylase; CitA, minor citrate synthase; CitZ, citrate synthase II; CitB, aconitase; Icd, isocitrate dehydrogenase; OdhA, 2‐Oxoglutarate dehydrogenase (E1 subunit); OdhB, 2‐Oxoglutarate dehydrogenase complex (dihydrolipoamide transsuccinylase, E2 subunit); PdhD, dihydrolipoamide dehydrogenase E3 subunit; SucC, succinyl‐CoA synthetase (beta subunit); SucD, succinyl‐CoA synthetase (alpha subunit); SdhA, succinate dehydrogenase (flavoprotein subunit); SdhB, succinate dehydrogenase; SdhC, succinate dehydrogenase (cytochrome b558 subunit); FumC, fumarase; Mdh, malate dehydrogenase; YhfS, hydroxymethylglutaryl CoA synthase; Dxs, 1‐deoxy‐d‐xylulose‐5‐phosphate synthase; IspC, 1‐deoxy‐d‐xylulose‐5‐phosphate reductoisomerase; IspD, 4‐diphosphocytidyl‐2‐C‐methyl‐d‐erythritol synthase; IspE, 4‐diphosphocytidyl‐2‐C‐methyl‐d‐erythritol kinase; IspF, 2C‐methyl‐d‐erythritol 2,4‐cyclodiphosphate synthase; IspG, 1‐hydroxy‐2‐methyl‐2‐(E)‐butenyl 4‐diphosphate synthase; IspH, 1‐hydroxy‐2‐methyl‐butenyl 4‐diphosphate reductase; Idi, isopentenyl pyrophosphate isomerase; IspA, garnesyl diphosphate synthase; Metabolite abbreviations: G6P, glucose‐6‐phosphate; F6P, fructose‐6‐phosphate; FBP, fructose 1,6‐bisphosphate; DHAP, dihydroxyacetone phosphate; G3P, glyceraldehyde‐3‐phosphate; G13P, glycerate 1,3‐diphosphate; 3‐PG, glycerate 3‐phosphate; 2‐PG, glycerate ‐2‐phosphate; PEP, phosphoenolpyruvate; DXP, 1‐deoxy‐d‐xylulose 5‐phosphate; MEP, 2‐C‐methyl‐d‐erythritol 4‐phosphate; CDP‐ME, 4‐(cytidine 5'‐diphospho)‐2‐C‐methyl‐d‐erythritol; CDP‐MEP, 2‐phospho‐4‐(cytidine 5'‐diphospho)‐2‐C‐methyl‐d‐erythritol; MEcDP, 2‐C‐methyl‐d‐erythritol 2,4‐cyclodiphosphate; HMBDP; 1‐hydroxy‐2‐methyl‐2‐butenyl 4‐diphosphate; IDP, isopentenyl diphosphate; DMADP, dimethylallyl diphosphate; GDP, geranyl diphosphate; FDP, farnesyl pyrophosphate; GGDP, geranylgeranyl pyrophosphate; HEPP, heptaprenyl diphosphate; UDPP, undecaprenyl diphosphate. [Colour figure can be viewed at wileyonlinelibrary.com]

*Bacillus subtilis* is known for high emission of isoprene compared to other species of bacteria including *E. coli* (Kuzma *et al*.^1995^). Isoprene, a simple form of a terpenoid molecule (also known as hemiterpene), is hypothesized as one of the signal molecules indicating the carbon metabolism rate of individual bacterium (Sivy *et al*.^2002^). Isoprene might also be the channel for the bacterium to drain out the terpenoids building blocks after some excess metabolism, in order to prevent further toxicity caused by prenyl diphosphate precursors such as dimethylallyl diphosphate (DMADP), isopentenyl diphosphate (IDP) or farnesyl diphosphate (FDP) (Sivy *et al*.^2011^). *Bacillus subtilis* has an endogenous methylerythritol phosphate (MEP) pathway to produce terpenoid building blocks, IDP and DMADP (Fig. 1).

As a Gram‐positive model bacterium, *B. subtilis* does not reflect the whole Gram‐positive terpenoid biosynthesis pathway. Gram‐positive cocci bacteria together with *Lactobacillus* own solely the mevalonate (MVA) pathway, while *Listeria* genera and a minor number of Actinobacteria such as *Streptomyces* own the MVA pathway as their secondary route in addition to MEP pathway (Lange *et al*.^2000^; Wilding *et al*.^2000^; Hedl *et al*.^2002^; Kuzuyama and Seto 2003; Campobasso *et al*.^2004^; Lombard and Moreira 2011; Heuston *et al*.^2012^). Meanwhile, most gram‐positive rod bacteria including *B. subtilis* possess the MEP pathway (Fig. 1) (Fall and Copley 2000; Wagner *et al*.^2000^).

The MEP pathway consists of eight enzymatic steps starting with the conjugation of pyruvate and glyceraldehyde 3‐phosphate (G3P) that eventually ends with DMADP and IDP as the universal precursors of terpenoids. Understanding the structure and biochemical properties of each enzyme and their respective reaction mechanisms would be ideal for performing further optimizations. Up until now, only three enzymes of *B. subtilis* MEP pathway have been structurally elucidated. Nevertheless, crystal structures of MEP pathway enzymes from other related micro‐organisms can be used as models in engineering *B. subtilis* enzymes.

## First step of the MEP pathway: Linking the Terpenoid and central carbon pathway

A functional study on the MEP pathway revealed that step 1 and 2 of MEP pathway are critical and that a reduction in gene expression of both constitutes enzymes which hampered the growth of the bacterium (Julsing *et al*.^2007^). Improvement of terpenoid production via MEP pathway usually starts with the overexpression of these two enzymes (Xue and Ahring 2011; Zhou *et al*.^2013^; Xue *et al*.^2015^). Hence, investigations on the enzyme structures and mechanisms of the reactions would be beneficial for improving the overall terpenoid production.

Dxs is responsible for the first step of the MEP pathway, the formation of 1‐deoxy‐d‐xylulose 5‐phosphate (DXP) from pyruvate and G3P. DXP is not only precursor for subsequent MEP pathway reaction but also for thiamine (vitamin B1) and pyridoxol (vitamin B6) biosynthesis (Hill *et al*.^1989^; Wagner *et al*.^2000^; Hazra *et al*.^2009^). Several studies indicate that formation of DXP is the limiting step of the MEP pathway (Julsing *et al*.^2007^; Zhao *et al*.^2011^; Banerjee *et al*.^2013^; Hess *et al*.^2013^; Banerjee and Sharkey 2014; Kudoh *et al*.^2017^). Suppression of the gene impaired the growth of the bacterium shown by its small colony and reduced isoprene emission and full suppression of the gene led to lethality (Julsing *et al*.^2007^). Meanwhile overexpression of the gene increased the isoprene emission (Hess *et al*.^2013^).

Dxs requires the presence of thiamine diphosphate (ThDP) as the cofactor. The requirement of ThDP is one of the properties shared by the transketolase group of enzymes including transketolases, the tricarboxylic acid (TCA) cycle and pentose phosphate pathway. Other enzymes which require ThDP include pyruvate decarboxylase that breaks down pyruvate forming acetaldehyde, pyruvate dehydrogenase and α‐ketoglutarate dehydrogenase of Kreb’s cycle (Xiang *et al*.^2007^; Kowalska and Kozik 2008). ThDP assists the binding of pyruvate in the active site of the enzyme by forming C2α‐lactylThDP (LThDP) (Patel *et al*.^2012^). LThDP bears a carbanion that is ready to contact with G3P. Upon G3P attachment, the 2‐hydroxyethyl moiety of pyruvate will ligate to the molecule and eventually lead to DXP formation accompanied by the release of CO_2_ (Patel *et al*.^2012^).

Negative feedback imposed by IDP and DMADP is natural to Dxs and exists across species. Nevertheless, a study comparing several different Dxs enzymes found that *B. subtilis* Dxs is more resistant to feedback inhibition compared to *E. coli* and other bacteria (Kudoh *et al*.^2017^). *Bacillus subtilis* Dxs is also considered to be more resistant to proteases as compared to Dxs from *E. coli*, *Paracoccus aminophilus* and *Rhodobacter capsulatus*.

### 2‐C‐methyl erythritol 4‐phosphate (MEP) production mediates forward reactions in MEP pathway

The second step of the MEP pathway involves the reduction and isomerization of DXP to produce MEP. It is speculated to involve two putative steps of a reaction catalyzed by 1‐deoxy‐d‐xylulose 5‐phosphate reductoisomerase (DXR), also known as IspC in microbes.

Dxr requires a divalent cation of Mg^2+^, Mn^2+^ or Co^2+^ and NADPH as cofactors. Mechanistic studies on Dxr suggested that divalent cation and NADPH should occupy their binding sites prior to the attachment of DXP. It is also showed that MEP could undergo the reverse reaction with the help of NADP^+^ resulting in DXP. However, this reverse reaction occurs at a very low rate and is limited by the presence of NADPH (Hoeffler *et al*.^2002^). Thus, the availability of NADPH ensures the forward reaction of DXP towards MEP.

The expression of both *B. subtilis* enzymes in *E. coli* led to more than twofold higher production of isoprene compared to *E. coli* strain overexpressing its own endogenous enzymes (Zhao *et al*.^2011^). While there is no available 3D structure of *B. subtilis* Dxr, other related micro‐organisms can be referred to for predicting the amino acid sequences involved in enzyme–substrate dynamic interaction.

### *B. subtilis* IspD facilitates efficient cytidyl transfer to MEP

In the following step, MEP obtains the additional cytidine monophosphate (CMP) moiety resulting in 4‐diphosphocytidyl‐2‐*C*‐methyl‐d‐erythritol (CDP‐ME) with the help of 4‐diphosphocytidyl‐2‐C‐methylerythritol synthase (CMS/IspD). The reaction requires cytidine triphosphate (CTP) as the donor of CMP, with a conjunct loss of pyrophosphate molecules.

IspD is in homodimeric conformation with each monomer containing up to 10 β sheets mostly in parallel configuration (Richard *et al*.^2001^). The enzyme possesses three loops that participate in binding and activity namely P‐loop, L1‐loop and L2‐loop. Hydrogen bonds among the amino acid residues inside the pocket play a role in the conformational change of the loop. In its inactive state, the loop is open and has more surface contact with the solvent. Upon the CTP‐Mg^2+^ binding, the pocket becomes narrower and there is less contact with the solvent. *Bacillus subtilis* IspD has narrower surface in contact with solvent compared to the *E. coli* version. This presumably has impact on the lower *K*_M_ of *B. subtilis* enzyme that eventually led to higher catalytic efficiency, that is, up to two folds of *E. coli* IspD (Jin *et al*.^2016^). The competition and interaction between solvent and CTP towards the pocket residues by hydrogen bond seems to be the primary cause. With more hydrogen bonds, the transition state would be more stable and readier for nucleophilic attack of MEP phosphate. With a higher catalytic efficiency, utilizing *B. subtilis* IspD would give extra flux on MEP pathway than in *E. coli*.

## IspE and IspF catalyze the formation of MECDP, acting as intermediates in the MEP pathway as well as oxidative‐stress response in bacteria

IspE is responsible for phosphate group addition to CDP‐ME molecule, generating 4‐diphosphocytidyl‐2‐C‐methyl‐d‐erythritol 2‐phosphate (CDP‐ME2P). IspE consists of two domains, ATP binding domain and substrate (CDP‐ME) binding domain (Kalinowska‐Tłuścik *et al*.^2010^). Volke *et al*. estimated that the amount of IspE is considered as the second highest amount of MEP enzymes, after IspH, in *E. coli* with a total maximum reaction rate up to 2·1 × 10^5^ molecules per min per cell (Volke *et al*.^2019^). In contrast, Dxs, IspF and IspG are estimated to have maximum reaction rate up to 16 × 10^3^, 6·66 × 10^3^ and 4·83 × 10^3^ molecules per min per cell respectively. Those three enzymes are considered as MEP pathway enzymes with low turnover numbers per cell. Hence compared to those three enzymes, IspE might not be considered as the limiting step of MEP pathway.

The subsequent reaction involves the cleavage of the cytidyl moiety and cyclization of CDP‐ME2P resulting in methyl erythritol cyclic diphosphate (MEcDP) catalyzed by IspF (Banerjee and Sharkey 2014). Hydrogen peroxide addition (up to 0·02%) into *B. subtilis* medium increased the isoprene emission up to twofolds (Xue and Ahring 2011; Hess *et al*.^2013^). It is suggested that MEcDP is involved in DNA stabilization upon the exposure to oxidative stress by preventing the peroxide formation (Artsatbanov *et al*.^2012^).

IspF presents in a homotrimer forming three active pockets with each situated at the interface of two vicinal monomers (Liu *et al*.^2018^). Compared to *E. coli*, *B. subtilis* IspF has a smaller solvent accessible surface that might influence the catalytic activity, but both possess hydrophobic cavity that is speculated to play a role in the binding of the inhibitor ligands (Bitok and Meyers 2012; Liu *et al*.^2018^). It is interesting to note that an *in vitro* study of *E. coli* IspF showed the stable complex formation between the enzyme and MEP, the product of Dxr/ IspC. The complex stabilized the enzyme activity and improved the catalytic efficiency by up to 4·8 times compared to IspF alone (Bitok and Meyers 2012). It is speculated that the improvement was facilitated by the higher affinity of the substrate, CDP‐MEP towards IspF. However, in contrast to IspF, IspF‐MEP complex is negatively affected by FDP and other prenyl diphosphate including DMADP and IDP. This might hold a regulatory mechanism to feedforward the MEP pathway but at the same time prevent the cell toxicity due to the prenyl phosphate build‐up. In another side, this fact is insightful in an effort to increase the MEP pathway flux. Increasing the supply of MEP would produce a domino effect by increasing the activity of IspF that ends up with higher supply of terpenoid precursor of IDP and DMADP. In addition, FDP should be utilized efficiently by the downstream pathway of terpenoid in order to prevent the feedback inhibition of FDP to IspF–MEP–CDP–MEP complex.

## The last two steps of MEP pathway involve reductive reactions

The last two steps of MEP pathway are reductive reactions. MEcDP conversion to 4‐hydroxy‐3‐ methylbut‐2‐enyl‐diphosphate (HMBDP) requires the cleavage of C‐O bond between the phosphate and C2 of the substrate. Meanwhile the last step of MEP pathway converts HMBDP to either IDP or DMADP by dehydroxylation and isomerization steps. In *E. coli*, both steps of MEP pathway require NADPH as the cofactor and flavodoxin/flavodoxin reductase (Wolff *et al*.^2003^; Gräwert *et al*.^2004^; Puan *et al*.^2005^). Mutation on *fldA* (encoding flavodoxin I) of *E. coli* decreased the HMBDP level dramatically, signifying the role of flavodoxin in the pathway. *Bacillus subtilis* owns two flavodoxins encoded by *ykuN* and *ykuP* and a ferredoxin (*fer*) in its genome (Lawson *et al*.^2004^; Girhard *et al*.^2010^). It also has ferredoxin (flavodoxin) reductase (*yumC*) (Seo *et al*.^2004^). However, the involvement of both flavodoxins or ferredoxin and their reductases in *B. subtilis* MEP pathway is still to be explored.

IspG and IspH are Fe‐S cluster containing enzymes, both of them are susceptible to reactive oxygen species and reactive nitrogen species. IspG forms homodimer, and each contains two domains (N and C domain) connected by a short linker of arginines (Lee *et al*.^2010^). The N domain of the enzyme contains the catalytic active site, while the C domain is responsible for Fe‐S cluster coordination. The reaction occurs at the interface of N domain from one monomer with the C domain from the other monomer (Liu *et al*.^2012^). The Fe‐S cluster is coordinated by three Cys and a Glu of the C domain and situated at the interface of both domains.

IspH is suspected to have promiscuous activities. In addition to having activity towards HMBDP, IspH isolated from alkaliphilic *Bacillus* sp. N16‐5 evidently possessed the isoprene and isoamylene synthase activity. Isoprene is generated from HMBDP, while two isoamylenes are directed from DMADP and IDP (Ge *et al*.^2016^). Yet, whether this activity is also found in *B. subtilis* 168 IspH still requires more exploration. In another *in vitro* study, IspH of *E. coli* was found to have acetylene hydratase activity catalyzing the conversion of acetylene into aldehyde or ketone (Span *et al*.^2012^). Nevertheless, this reaction took place on the oxidized IspH, underestimating its significance in the cytosol of the bacteria. The occurrence of these promiscuous events would underscore the divergence of MEP flux through IspH and its inhibition would lead to more IDP and DMADP.

## Isopentenyl diphosphate isomerase (Idi) balances the IDP and DMADP content

Methylerythritol phosphate pathway of *E. coli* is able to generate IDP and DMADP simultaneously approximately in a ratio of 1 : 5 (DMADP to IDP) (Rohdich *et al*.^2002^; Volke *et al*.^2019^). In contrast, the MVA pathway can only provide IDP from the decarboxylation of mevalonate diphosphate (the last step of the pathway) and therefore strictly requires Idi to provide DMADP (Dewick 2002). In *E. coli*, the transcript number of endogenous Idi is noticeably low and this might be due to its nonessential role under natural circumstance (Hahn *et al*.^1999^; Volke *et al*.^2019^). A study on conditional knock‐out of Idi also revealed its nonessentiality to *B. subtilis* growth (Julsing *et al*.^2007^).

In contrast to *E. coli* that possesses type I Idi, *B. subtilis* owns type II Idi which is phylogenetically closer to Gram‐positive bacteria that possess MVA instead of MEP pathway (Steinbacher *et al*.^2003^; Laupitz *et al*.^2004^). While type I Idi requires only divalent cations as the cofactor, type II Idi requires FMN and NADPH under aerobic conditions. It is also interesting to note that type II Idi has an l‐lactate dehydrogenase activity.

Dimethylallyl diphosphate constitutes only the head part of prenyl diphosphate, while IDP would be required for the addition of allyl group in prenyl diphosphate elongation/ condensation. Hence the longer the prenyl precursor of a certain terpenoid is, the lower DMADP/IDP ratio would be required. As an illustration, to generate one molecule of FDP as precursor of sesquiterpenes, it requires one1 molecule of DMADP and two molecules of IDP, while GDP (the precursor of monoterpenes) requires an equal mol of DMADP and IDP. Thus, the balance between IDP and DMADP of MEP pathway would be more significant for producing small terpenoids such as isoprene or monoterpenes than for large terpenoids such as carotenoids.

IDP or DMADP can undergo further rearrangements through dephosphorylation yielding hemiterpene (C5 terpenoid) like isoprene. In addition to isoprene, *B. subtilis* is also able to produce isopentenol and dimethyl allyl alcohol, the alcohol derivative of IDP and DMADP respectively. Generation of isopentenol or prenyl alcohol involves a specific DMADP/IDP phosphatase. NudF and YhfR, the two phosphatases of *B. subtilis* that belong to ADP‐ribose phosphatase superfamily, are responsible for the dephosphorylation of DMADP and IDP (Withers *et al*.^2007^; Li *et al*.^2018^).

## Isomerization and condensation of terpenoid precursors

Prenyl transferases catalyze the condensation reaction of IDP and DMADP resulting in GDP (monoterpene substrate, C10), FDP (sesquiterpenes substrate, C15), GGDP (diterpenes substrate, C20) or higher prenyl substrates such as heptaprenyl diphosphate (C35 terpene) or undecaprenyl diphosphate (C55 terpene). *ispA* gene of *B. subtilis* encodes FDP synthase, an enzyme for conjugation of two IDP and single DMADP molecules producing FDP. Some terpenoids are important for *B. subtilis* physiology and metabolism, for example, ubiquinone (important for electron transport), farnesol (an alcohol derivative of FDP important for the formation of biofilm), sporulene (a C35 terpene acting as antioxidant during the sporulation) (Bosak *et al*.^2008^; Kontnik *et al*.^2008^) and undecaprenyl diphosphate (a C55 terpene involves in cell wall biogenesis) (Noike *et al*.^2008^; Kingston *et al*.^2014^; Zhao *et al*.^2016^). Accumulation or depletion of essential endogenous terpenoid could be harmful for the bacterium. High formation of some prenyl diphosphates (IDP, DMADP and FDP) has been known to cause cellular toxicity (Martin *et al*.^2001^; Sivy *et al*.^2011^). Depletion of farnesol by knockout *yisP* prevents the bacterium to generate biofilms (Feng *et al*.^2014^). Meanwhile, overexpression of *hepT* and *hepS* to increase heptaprenyl diphosphate production could disrupt the cell wall biogenesis (Kingston *et al*.^2014^; Zhao *et al*.^2016^). Therefore, improvement on the production of terpenoids of economic importance should also consider the flux towards those essential endogenous terpenes.

## Metabolic engineering of *B. subtilis* for terpenoid cell factory

Well known for its capability to emit high amounts of isoprene, *B. subtilis* was expected to be a superior microbial platform for terpenoid production. Though the fact that developing *B. subtilis* is lagging behind compared to *E. coli* and *S. cerevisiae* due to late development of its molecular tools, recent studies on *B. subtilis* show very promising results to develop it into terpenoid cell factories (summarized in Table 2). Production of isoprene, carotenoids, amorphadiene, taxadiene and menaquinone‑7 (MK‐7) with various bioactivities have been explored and boosted in *B. subtilis*.

**Table 2:** Production of terpenoids by engineered *Bacillus subtilis*

Terpenoids	Classification	Strategy	Culture conditions	Titre/yield	Reference
Isoprene	Hemeterpenoids (C5)	Dxs was overexpressed	Shake‐flask fermentation	–	Xue and Ahring, (2011)
		Isoprene synthase (*kIspS*) gene overexpression	Shake‐flask fermentation	1434·3 μg l^−1^	Gomaa *et al*.(2017)
Amorphadiene	Sesquterpenoids (C15)	Amorphadiene synthase was fused with six arginine tag at N‐terminus, dxs and idi were overexpressed	Shake‐flask fermentation	20 mg l^−1^	Zhou *et al*.(2013)
Taxadiene	Diterpenoids (C20)	Geranylgeranyl diphosphate synthase (crtE) was overexpressed, all MEP pathway enzymes and ispA were overexpressed	Shake‐flask fermentation	17·8 mg l^−1^	Abdallah *et al*.(2019)
4,4′‐diapolycopene and 4,4′‐diaponeurosporene	Triterpenoids (C30)	crtMN was overexpressed in high‐copy number plasmid, all MEP pathway enzymes and ispA were overexpressed	Shake‐flask fermentation	10·65 mg g^−1^	Yoshida *et al*.(2009); Xue *et al*.(2015); Abdallah *et al*.(2020)
Squalene	Triterpenoids (C30)	Dxs, ispD, ispF, ispH and ispA overexpressed in high‐copy number plasmid	Shake‐flask fermentation	7·5 mg l^−1^	Song *et al*.(2020)
Menaquinone‐7	Terpenoid‐quinones (C35)	Overexpression of menA, dxs, dxr, yacM‐yacN, glpD and deletion of dhbB	2 l bioreactor fed‐batch fermentation	69·5 mg l^−1^	Yang *et al*.(2019)
		Overexpression of menA‐dxs‐dxr‐idi	Shake‐flask fermentation	50 mg l^−1^	Ma *et al*.(2019)
		Fine‐tuned expression of different modules by applying Phr60‐Rap60‐Spo0A quorum‐sensing molecular switch	Shake‐flask fermentation 15 l bioreactor fed‐batch fermentation	9–360 mg l^−1^ 200 mg l^−1^	Cui *et al*.(2019)

### Carotenoids

Carotenoids are being widely used in food, pharmaceutical and health protection industries. Early metabolic engineering on *B. subtilis* utilized two genes from *Staphylococcus aureus* (*crtM* and *crtN*) involved in the biosynthesis of C30 carotenoids especially 4,4′‐diapolycopene and 4,4′‐diaponeurosporene (Yoshida *et al*.^2009^). Relying only on the endogenous MEP pathway with a constitutive promoter regulating the expression of *crtM* and *crtN*, engineered *B. subtilis* could produce C30 carotenoids that lead to a higher resistance to oxidative stress exemplified with H_2_O_2_ (Yoshida *et al*.^2009^). However, there was no report on the quantity of the carotenoid product. Later work on engineering *B. subtilis* was directed at higher isoprene production and at the same time focusing on the most influential gene of the endogenous MEP pathway. Overexpression of *dxs,* but not *dxr,* leveraged isoprene emission of *B. subtilis* especially at the early and middle logarithmic phase (Xue and Ahring 2011). Meanwhile, modification of the medium by adding more salt, hydrogen peroxide and also heating up to 40°C increased the release of isoprene.

To further improve terpenoid production, overexpression of multiple MEP pathway genes was found to increase C30 terpenoid production in *B. subtilis* (Xue *et al*.^2015^). Xue *et al*.(2015) cloned MEP pathway genes step by step into two different constructs resulting in two strains of *B. subtilis* with each operon consisting of four enzymes of the MEP pathway, that is, SDFH subset for *dxs‐ispD‐ispF‐ispH* operon and CEGA subset for *ispC/dxr‐ispE‐ispG‐ispA* operon. As the read out, Xue et al utilized *crtM* and *crtN* genes encoding two enzymes involved in C30 carotenoid production. It is quite surprising that the strains with upregulation of *dxr/ispC* could produce high level of C30 carotenoid comparable to, if not better to strains overexpressing *dxs*. Eventually, the two strains with two different subsets of artificial operon as mentioned above could and can produce C30 carotenoid at more than 15‐fold increase (910 mg g^−1^ dcw) compared to *B. subtilis* carrying only the genes for carotenoid production (0·6 mg g^−1^ dcw). Interestingly, in another experiment, overexpression of *dxr* alone did not bring improvement to the isoprene production (Xue and Ahring 2011). These results can be explained by the high flux into the carotenoid pathway resulting in actual low levels of DMADP or IDP preventing negative feedback. In our recent result, upregulating the whole MEP pathway has further thrived the carotenoid production by up to around 20 mg g^−1^ dcw (Abdallah *et al*.^2020^), which was twofold higher compared to our previous result with only four enzymes of MEP pathway being upregulated.

### Amorphadiene

Artemisinin is a sesquiterpene lactone which is by far the most effective antimalarial drug. Converting the precursor amorphadiene produced by microbes through chemical methods to artemisinin is considered to be more attractive than directly extracting from its host plants. Researchers have tried to construct the amorphadiene biosynthesis pathway in *B. subtilis*. Co‐expression of amorphadiene synthase (ADS) with *dxs* and *idi*, yields around 20 mg l^−1^ of amorphadiene in flask scale (Zhou *et al*.^2013^). Dxs performs the first enzymatic step of MEP pathway that considered the determinants of the pathway (Volke *et al*.^2019^). Meanwhile, Idi acts as an IDP isomerase converting IDP to DMADP or vice versa. In MVA pathway, Idi is essential as the final step of the pathway only produces IDP from decarboxylation of diphosphomevalonate. Hence, Idi is very critical in balancing the high flux of IDP generated by the MVA pathway. In contrast, the MEP pathway inherently produces both terpenoid precursors in parallel and therefore Idi overexpression probably is not essential. The high expression of ADS is mandatory in order to maximize the utilization of prenyl precursors. With respect to the negative feedback from prenyl precursors IDP, DMADP, GDP or FDP to Dxs, a high flux of the MEP pathway gives no benefit unless the downstream part of the pathway can utilize the provided precursors efficiently (Banerjee *et al*.^2013^). Improving ADS translation by modifying the N‐terminus of the protein proved to increase the amorphadiene production by up to 2·5‐fold (Zhou *et al*.^2013^). It is also interesting to note that a high flux of prenyl precursors, such as FDP, might be toxic to the cells implying the importance of higher expression of active terpene synthases (Martin *et al*.^2003^; Sivy *et al*.^2011^). N‐terminal fusion of green fluorescent protein to ADS significantly improved the expression of ADS and led to better production of amorphadiene. Providing more supply of precursors by additional expression of IspA and whole MEP pathway improved the production by up to 42·5 mg l^−1^. With medium modification by additional pyruvate and K_2_HPO_4_, our recent result shows very promising capacity of *B. subtilis* to produce this antimalarial artemisinin precursor (416 mg l^−1^) (in submission).

### Taxadiene

Taxadiene is the critical precursor of the well‐known anticancer drug paclitaxel (Taxol®). Functional production of taxadiene in *B. subtilis* was attained by combining the heterologous expression of taxadiene synthase (TXS) in combination with the regulated overexpression of the full MEP pathway including *ispA*, the FDP synthase encoding gene. Overexpession of *B. subtilis ispA* did not lead to the production of taxadiene, suggesting that IspA does not act as the geranyl geranyl diphosphate synthase. Co‐expression of *crtE* (the GGDPS encoding gene of *Pantoea ananatis*) together with the synthetic operon of MEP pathway and TXS resulted in 17·8 mg l^−1^ of taxadiene in *B. subtilis* (Abdallah *et al*.^2019^). This surpasses the result achieved in yeast (8·7 mg l^−1^) (Engels *et al*.^2008^). Higher amounts of taxadiene were achieved by fine tuning the expression of MEP pathway in *E. coli* leading to 1 g l^−1^ of the product in fed‐batch fermentation (Ajikumar *et al*.^2010^). Taking this result as an inspiration, further improvement on *B. subtilis* taxadiene production capability might involve fine tuning the MEP pathway genes through different strengths of promoters or ribosome binding sites (RBS).

### Menaquinone‑7

MK‐7, belonging to terpenoid‐quinones, is the major vitamin K2 compound, being extensively applied for promoting bone growth and cardiovascular health. Previously, many *B. subtilis natto* strains have been screened and mutated to produce MK‐7 by traditional fermentation without genetic modification (Mahdinia *et al*.^2018^). Recently, *B*. *subtilis* 168 was employed as chassis cells to produce and increase biosynthesis of MK‐7 by modular pathway engineering (Yang *et al*.^2019^). Four endogenous modular pathways (MK‐7 pathway, shikimate pathway, MEP pathway and glycerol metabolism pathway) are related to the biosynthesis of MK‐7, and parent strain could produce 3·1 mg l^−1^ MK‐7. When *menA* (MK‐7 pathway) were overexpressed under promoter P*laps*, 2·1‐fold MK‐7 yield compared to the starting strain could be obtained. Also, simultaneous overexpression of four MEP pathway genes (*dxs, dxr/ispC, yacM/ispD and yacN/ispF*) together with *menA* led to 12·0 mg l^−1^ of MK‐7. With a further enhancement of the glycerol metabolism by overexpressing *glpD* and decreasing the intermediate metabolite consumption by *dhbB* knockout, the final production of MK‐7 significantly increased to 69·5 mg l^−1^ after 144 h of fermentation.

Interestingly, the integration sites for overexpression of MEP pathway genes also affect the final production of MK‐7. Based on *Bacillus* minimum genome, Yang et al inserted *menA, dxs* and *dxr* into three different loci: *yxlA, yjoB* and *ydeO*, respectively (Yang *et al*.^2019^). However, when *menA‐dxs‐dxr‐idi* were placed at the *amyE* locus of *B. subtilis* as an operon under IPTG‐inducible promoter P*spac*, the final titre of MK‐7 significantly increased to 50 mg l^−1^ without further optimization (Ma *et al*.^2019^). Their results also indicated that overexpression of *idi* was beneficial in the presence of *menA*, *dxs* and *dxr*. To further improve the production of MK‐7, dynamically balanced cell growth and target compound synthesis is necessary. Cui *et al*.(2019) constructed the Phr60‐Rap60‐Spo0A quorum‐sensing molecular switch, which could dynamically upregulate and downregulate the expression level of related pathways without adding any inducers. Thus, the MK‐7 production level increased from 9 to 360 mg l^−1^ in *B. subtilis*, which is by far the highest production level reported at flask incubation level.

## Current genetic engineering tools and promising strategies to improve terpenoid production in *B. subtilis*

Current engineering on *B. subtilis* for terpenoid cell factory still relies on the limited number of replicative plasmids as vector. Replicative plasmids are easier to handle and possess higher flexibility for expression manipulation. Based on replication mode, there are two types of plasmids, rolling circle replicating and theta replicating plasmids. Majority of *B. subtilis* plasmids, especially for high copy number plasmids, belong to rolling circle plasmids. However, rolling circle plasmids suffer from instability, especially those with more than 10 kilo base pairs of inserts. Theta replication plasmids offer more stability than rolling circle plasmids, but natural theta plasmids of *B. subtilis* are quite rare and mostly have large sizes (more than 50 kbps) (Meijer *et al*.^1998^). Nonetheless, several theta replication plasmids are currently available with different origins of replication allowing them to be combined (Nguyen *et al*.^2005^; Popp *et al*.^2017^).

In contrast to laboratory scale, fermentation at industry requires highly stable microbial strains. Integrative plasmids would be more acceptable as the gene would be integrated to the bacteria chromosome. Currently there is a bacillus tool box providing different types of promoters, RBSs and integrative plasmids for *B. subtilis* (Radeck *et al*.^2013^). Engineering on RBSs and constitutive promoters of *B. subtilis* has made it possible to tune protein expression by five orders of gradients (Guiziou *et al*.^2016^; Castillo‐Hair *et al*.^2019^). At genomic level, various manipulation tools for replacing or eliminating genes are also available (Wang *et al*.^2012^; Dong and Zhang 2014; Toymentseva and Altenbuchner 2019). Current CRISPR/Cas9 toolkit for *B. subtilis* has high efficiency and precision (Toymentseva and Altenbuchner 2019). Toxin–antitoxin system consisting of EndoA‐EndoB has been employed for protein expression in *B. subtilis* without the need of antibiotics as selective agents (Yang *et al*.^2016^). These might serve as beneficial tools either for nonnative gene insertion or fine‐tuning expression of particular genes of *B. subtilis*.

Another requirement on optimum expression of nonnative protein is codon optimization. *B. subtilis* owns three different classes of genes based on the codon preference. Class I with weak preference constitutes mainly genes involved in the intermediary metabolism, meanwhile class II has a very strong preference and constitutes genes responsible for exponential growth of the bacterium (Moszer *et al*.^1999^). Class III has its different properties with A+U rich codon preference that mostly belong to horizontally transferred gene (Moszer *et al*.^1999^). Nonetheless, compared to *E. coli*, *B. subtilis* has less bias on codon usage (Shields and Sharp 1987). This implies that codon optimization might have less relevant benefits for heterologous protein expression in *B. subtilis*.

As mentioned in previous section, *B. subtilis* could emit high amount of isoprene. With current genetic tools, there are more options in modulating terpenoid pathway at the genetic level. Flux improvement of the pathway evidently improved the production of several valuable terpenoids in *B. subtilis* including amorphadiene, carotenoids, taxadiene and menaquinones.

### Protein engineering

Further efforts to increase terpenoid production might also involve protein engineering. Upregulating the expression of an enzyme or a pathway costs high energy for the cell replication, transcription and translation of particular proteins (Lynch and Marinov 2015). This high energy cost could be reduced by trade‐off between the expression level and enzyme catalytic activity. In addition, protein engineering could also be a tool to eliminate certain inhibition events by substrates or products or to eliminate unwanted side products (Hult and Berglund 2007).

Currently, there is still a small effort in protein engineering of the MEP pathway enzymes. Dxs for example has been a subject of site directed mutagenesis for alleviating the negative feedback inhibition of IDP/ DMADP. Mutation at A147G/A352G of *P. trichocarpa* Dxs which involves in the binding of IDP, reduced IDP binding affinity slightly (Banerjee *et al*.^2016^). However, it came with cost of higher *K*_M_ of ThDP and pyruvate that overall decreased the catalytic efficiency of the enzyme about 15 times compared to the wild type (Banerjee *et al*.^2016^). *Bacillus subtilis* Dxs has been found to be more resistant to negative feedback of IDP/ DMADP but it has higher *K*_M_ compared to Dxs of *E. coli* (five times higher for G3P and three times higher for pyruvate) (Kudoh *et al*.^2017^). Yet, expression of *B. subtilis* Dxs in *E. coli* produced higher amount of isoprene compared to Dxs of other micro‐organisms including *E. coli* counterpart after 24 h of incubation. The mechanism of *B. subtilis* Dxs resistant to negative feedback is still elusive since the binding site of ThDP is generally homologous. Apart from unsuccessful effort on engineering negative feedback‐resistant Dxs, single amino acid mutation on Dxs of *E. coli* and *D. radiodurans* has been found to increase their catalytic activities. Mutation on Y392F of *E. coli* Dxs increased the relative catalytic activity by more than 2·5‐fold compared to the WT (Xiang *et al*.^2007^). It is suggested that Y392 indirectly involves in the binding of G3P and with the alteration to Phe gave more optimum space for G3P to interact with ThDP.

As mentioned earlier, IspF (in addition to Dxs and IspG) is considered as MEP pathway enzymes with low maximum reaction rate per cell in *E. coli* (Volke *et al*.^2019^). *In vitro* experiment showed that IspF is subject to both positive and negative feedbacks by MEP (the second intermediate product of MEP pathway) and FDP respectively (Bitok and Meyers 2012). It comes as the effect of inhibition of the MEP‐IspF complex which helps the enzyme to bind to CDP–MEP as the substrate. Engineering IspF with FDP resistant property would be another way to enhance the MEP pathway capacity.

Not only to MEP pathway, protein engineering could also be applied to terpene synthases. Site directed mutagenesis to improve catalytic activity has been performed on ADS, and levopimaradiene synthase (LPS), the enzyme responsible for generating a diterpene precursor of ginkgolides. *Escherichia coli* expressing M593I mutant of LPS increased the overall productivity up to 3·7‐fold compared to the bacterium with WT LPS (Leonard *et al*.^2010^). Meanwhile, double mutant variant (M593I/Y700F) showed productivity of 10 fold higher than WT with no production of abietadiene as one of the side products of LPS. One of the characteristics of terpene synthase is its promiscuity that causes the enzyme to produce multitude minor products. Promiscuity would direct the flux not only to the major product but also to minor products which causes the inefficiency. This could also hamper the subsequent purification of the products with quite close physicochemical properties. Another example is the double mutant of ADS (T399S/H448A) that was evidently four times more efficient than the WT though with a slightly higher *K*_M_ to FDP (Abdallah *et al*.^2018^). Overall productivity showed that *E. coli* expressing double mutant ADS produced amorphadiene three times higher than WT after 24 h of incubation. At the end, combining the highly active terpene synthase with upregulated isoprenoid precursor pathway (either MVA or MEP pathway) would be a potential approach on optimizing bacterial terpenoid cell factory, including *B. subtilis*. However, the structural elucidation or modelling of the specific enzymes would be necessary.

Downstream of the terpenoid pathway often involves hydroxylation or oxidation in general, and requires the involvement of specific monoxygenease P450s. Paclitaxel (Taxol®) requires eight specific P450s for specific oxygenation steps (Biggs *et al*.^2016^). Meanwhile amorphadiene conversion to dihydroartemisinic acid, a close precursor of artemisinin, involves a specific CYP450 called CYP71AV1 of *Artemisia annua* (Covello 2008). Eukaryotic expression of CYP450s in bacteria is often problematic as they are generally membrane bound proteins. In fact, this problem is hampering the use of bacterial terpenoid cell factory for further steps of terpenoid production. Several microbial cytochromes have been known for their capability on hydroxylation of terpenes. CYP109B1 of *B. subtilis,* for example, has the ability to oxidize valencene to nootkatone (a sequiterpene with grape fruit fragrance) (Girhard *et al*.^2009^, ^2010^). CYP102A1 of *Bacillus megaterium* (aka. P450BM3) has been known as one of the most versatile bacterial cytochromes (Whitehouse *et al*.^2012^). CYP102A1 has been extensively engineered including for amorphadiene oxidation. Tetramutant variant of P450BM3 was able to convert amorphadiene to amorphadiene epoxide up to 250 mg l^−1^ in *E. coli* (Dietrich *et al*.^2009^). This amorphadiene epoxide then underwent four chemical synthesis steps to yield dihydroartemisinic acid as the closest precursor of artemisinin. Up until now, cytochrome‐mediated steps of terpenoid biosynthesis is still one of the challenges in using bacteria as the platform. More exploration on bacterial cytochromes capable on terpene functionalization would definitely facilitate the advancement on engineering and utilization of bacterial terpenoid cell factory including *B. subtilis*.

### Heterologous MVA pathway

The MVA heterologous pathway expression might also be considered when performing metabolic engineering of *B. subtilis* as a metabolite cell factory. MVA pathway has been known long before the MEP pathway and was discovered almost three decades ago. Both eukaryotic and prokaryotic organisms can be the genetic sources of a heterologous MVA pathway. Several prokaryotes, as has been described at the beginning of the chapter, depend on MVA‐ rather than MEP‐ pathway for the production of terpenoid precursors. Heterologous MVA pathway might offer a less strict regulation at genetic levels as well as possible allosteric interactions with the existing cellular pathways. Still, some issues regarding the interconnectedness between its metabolites especially at the pathway upstream to the central carbon metabolism should not be underestimated. Notwithstanding, the pathway has been successfully expressed in *E. coli* to produce amorphadiene by up to 700 mg l^−1^ in flask scale after 48 h of incubation and 29 g l^−1^ (100 h of incubation) in fed batch fermentation after adjustments of metabolites flux (Tsuruta *et al*.^2009^; Ma *et al*.^2011^).

### Cofactor regenerating system

One of the important strategies in pathway optimization is cofactor supply. Both MEP and MVA pathway require NADPH as the electron carriers involved in reductive reactions. The cofactor is also required in redox reactions facilitated by CYP450s in many terpene functionalization. NADPH is involved in most of the anabolic cellular reactions and thus competition would be present whenever the terpenoid pathway flux is pushed. Thus, regeneration system to sustain NADPH supply is required. Many NADPH regenerating modules have been employed to support high titre metabolite production. Upregulating the expression of *zwf* encoding glucose‐6‐phosphate dehydrogenase has been utilized in the *Bacillus* genus such as for riboflavin (Duan *et al*.^2010^; Wang *et al*.^2011^), poly‐γ‐glutamic acid production (Cai *et al*.^2017^) and bacitracin (Zhu *et al*.^2019^). However, upregulating pentose phosphate pathway would split the glucose utilization which will decrease ATP and/or acetyl‐coA production. Other approaches include expression of heterologous NADH kinase (POS5) of *S. cerevisiae* to phosphorylate NADH (Lee *et al*.2013a, 2013b) and replacement of the native NAD^+^‐dependent glyceraldehyde 3‐phosphate by NADP^+^‐dependent dehydrogenase (GAPDH) facilitated by GapC of *Clostridium acetobutylicum* (Martínez *et al*.^2008^; Lee *et al*.2013b) or GapB of *B. subtilis* (Wang *et al*.^2013^). *gapA* substitution to *gapC* significantly increased lycopene and caprolactone production in *E. coli* but lower metabolite flux to pentose phosphate pathway. Upregulation of *pos5* and *zwf* significantly improved lycopene production in *S. cerevisiae* (Zhao *et al*.^2015^). However, in other experiments to promote production of protopanaxadiol, precursor of ginsenoside, in baker yeast, *pos5* overexpression resulted in decreased cell growth and eventually lower production of the compound (Kim *et al*.^2018^). Kim et al improved protopanaxadiol production in *S. cerevisiae* with more global approaches, by deleting *zwf*, replacing *ald2* encoding NAD^+^‐dependent acetaldehyde dehydrogenase with NADP^+^‐dependent isoform *ald6,* and replacing *gdh1*, encoding NADPH‐dependent glutamate dehydrogenase with NADH‐dependent isoform *gdh2* (Kim *et al*.^2018^). *zwf* overexpression though supplies more NADPH, also decreased the production of protopanaxadiol as the competition for pentose phosphate with glycolysis pathway.

NADPH involvement in anabolic pathway renders stricter regulation than NADH (Grabowska and Chelstowska 2003; Kim *et al*.^2018^). Hence, replacing the NADPH‐dependent HMGR by NADH‐dependent counterpart would compromise the trickiness in cofactor regeneration. Ma *et al*. exploited HMGR of *D. acidovorans* in which the organism is consuming NADH instead of NADPH in combination with overexpression of formate dehydrogenase (FDH) of *Candida boidinii*. Formate supplementation into the medium considerably increased amorphadiene production (Ma *et al*.^2011^). Meanwhile, Meadows *et al*.(2016) replaced yeast HMGR with NADH‐dependent HMGR of *Silicibacter pomeroyi* together with higher supply of acetyl‐coA, and enabled *S. cerevisiae* to produce up to 130 g l^−1^ farnesene in bioreactor scale.

## Further strategies and conclusion

**Figure 2:**
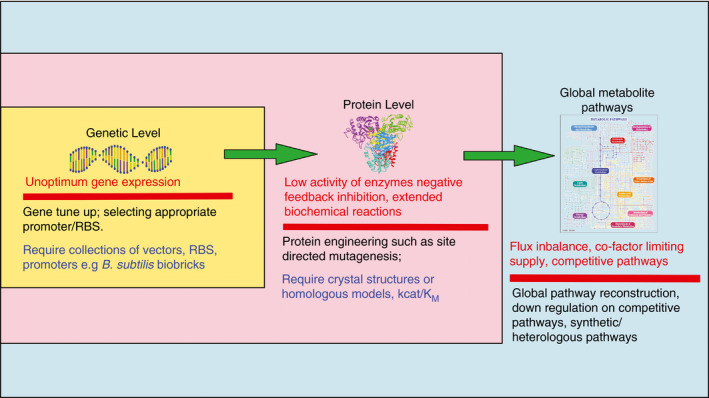
A systematic approach for optimum metabolic engineering of *Bacillus subtilis*. The traditional approach involves fragment optimization including manipulation of genetic expression cassettes or protein engineering enzymes of the pathway (innermost frame). Selection of promoter and RBS would be required at this step. Protein engineering assists obtaining enzymes with desired catalytic activities (middle frame). Taking further, optimization might involve the flux tuning and manipulation on proximal biochemical process including co‐factor supply. In a comprehensive optimization process, the multilayer Omics analysis is required by combining information from genomics, transcriptomic, proteomics and metabolomics data (outmost frame). [Colour figure can be viewed at wileyonlinelibrary.com]

*Bacillus subtilis* has become a potential microbial platform for high production of valuable terpenoids. Some inherent tools of *B. subtilis* such as many potential CYP450s and glycosyltransferases would accentuate further utilization of the bacterium for diverse terpenoids. Current development on molecular tools of *B. subtilis* provides stepping stones for more comprehensive measurements and engineering. One of the critical steps is to understand well the characteristics of each enzyme in the biosynthetic pathway and their complicated regulations. With limited information of steady state kinetic parameters of each enzyme of its endogenous terpenoid pathway, currently available engineering on *B. subtilis* still focuses on overexpression of genetic elements of the pathway. Fine‐tuning of multiple enzymes of a pathway is currently possible with a diverse selection of promoters and RBSs available for *B. subtilis* (Fig. 2).

While gene expression manipulation could be the main approach in metabolic engineering, upregulation of a gene is energy costly (Lynch and Marinov 2015). This implicates that manipulation on genes expression of a pathway would further burden the cells. Protein engineering such as by directed evolution approach would be an entry point to elevate the catalytic activity of certain enzymes or have more control by reducing the negative feedback inhibition (Banerjee *et al*.^2013^). Other subjects of protein engineering could also cover the catalytic activity focusing. Promiscuity is typical to terpene synthases leading to distribution of certain amount of terpene precursors into main and several minor products. Reducing the promiscuity of the enzymes would streamline the utilization of the precursors that at the end would reduce in total metabolic burden of the cells. Other approaches in protein engineering might also involve protein fusions and synthetic protein scaffolds. Both approaches are aimed to direct the enzyme in proximity to the precursors or cofactor supply. In some micro‐organisms such as *Campylobacter jejuni* and *Agrobacterium tumefaciens*, the sequential precursors and products of IspD, IspE and IspF are channelled by natural scaffolding of those enzymes. Synthetic scaffolding of MVA pathway enzymes has been utilized to elevate terpenoid production in *E. coli* (Dueber *et al*.^2009^). Meanwhile protein fusion has been one of approaches to improve the expression, solubility and stability of particular enzymes. It has also been utilized to attach the flavodoxin and flavodoxin reductase thus providing improved coupling efficiency to support CYP450 activity (Bakkes *et al*.^2015^).

Taking the perspective into cellular level, expression manipulation of certain genetic elements or protein engineering of particular enzymes of the terpenoid pathway could have a wider impact not only on the pathway itself but also on other biochemical processes (Hess *et al*.^2013^; Guan *et al*.^2015^). Several issues such as insufficient of NADPH or ATP or other cofactors and accumulated toxic intermediates are among the problems generally faced after pathway upregulation. Distal‐related biochemical pathway could also be hampered. For example, imbalanced heterologous expression of MVA pathway in *E. coli* perturbed the fatty acid metabolism leading to toxicity. As a result, the cellular productivity could be far from optimum. A more holistic view involving multilevel engineering including gene expression manipulation, protein engineering and followed by sophisticated multilayer Omics data capable on dissecting the implications at the genetic, protein and metabolites level would be necessary to give a comprehensive picture (Fig. 2) (Zhao *et al*.^2013^). Based on these data, flux constraint and limiting factors can be mapped and modelled that guide further integrated optimization involving multibiochemical process and genome wide regulation. At this point, genomic engineering tools such as CRISPR‐Cas or other multiplexed genomic engineering become essential. These comprehensive approaches will no doubt become essential processes for having an optimum strain for valuable terpenoids or secondary metabolites in general.

## Author contribution

H.P. and W.Q. conceived the idea of review. H.P. and Y.S. wrote the initial draft of the manuscript. W.Q. provided specific comments, edited and improved the manuscript. E.Y. and S.S. contributed to the various revisions of the manuscript. All the authors read and approved it for publication.

## Conflict of Interest

None declared.

## References

[jam14904-bib-0001] Abdallah, I.I. , van Merkerk, R. , Klumpenaar, E. and Quax, W.J. (2018) Catalysis of amorpha‐4,11‐diene synthase unraveled and improved by mutability landscape guided engineering. Sci Rep 8, 9961. 29967474 10.1038/s41598-018-28177-4 PMC6028579

[jam14904-bib-0002] Abdallah, I.I. , Xue, D. , Pramastya, H. , van Merkerk, R. , Setroikromo, R. and Quax, W.J. (2020) A regulated synthetic operon facilitates stable overexpression of multigene terpenoid pathway in *Bacillus subtilis* . J Ind Microbiol Biotechnol 47, 243–249. 31894423 10.1007/s10295-019-02257-4

[jam14904-bib-0003] Abdallah, I.I. , Pramastya, H. , van Merkerk, R. , Sukrasno, S. and Quax, W.J. (2019) Metabolic engineering of *Bacillus subtilis* toward taxadiene biosynthesis as the first committed step for taxol production. Front Microbiol 10, 218–227. 10.3389/fmicb.2019.00218 30842758 PMC6391936

[jam14904-bib-0004] Ajikumar, P.K. , Xiao, W.‐H. , Tyo, K.E.J. , Wang, Y. , Simeon, F. , Leonard, E. , Mucha, O. , Phon, T.H. *et al*. (2010) Isoprenoid pathway optimization for taxol precursor overproduction in *Escherichia coli* . Science 330, 70–74. 20929806 10.1126/science.1191652 PMC3034138

[jam14904-bib-0005] Artsatbanov, V.Y. , Vostroknutova, G.N. , Shleeva, M.O. , Goncharenko, A.V. , Zinin, A.I. , Ostrovsky, D.N. and Kapreliants, A.S. (2012) Influence of oxidative and nitrosative stress on accumulation of diphosphate intermediates of the non‐mevalonate pathway of isoprenoid biosynthesis in corynebacteria and mycobacteria. Biochemistry 77, 362–371. 22809155 10.1134/S0006297912040074

[jam14904-bib-0006] Bakkes, P.J. , Biemann, S. , Bokel, A. , Eickholt, M. , Girhard, M. and Urlacher, V.B. (2015) Design and improvement of artificial redox modules by molecular fusion of flavodoxin and flavodoxin reductase from *Escherichia coli* . Sci Rep 5, 10.1038/srep12158. PMC4503991 26177696

[jam14904-bib-0007] Banerjee, A. , Preiser, A.L. and Sharkey, T.D. (2016) Engineering of recombinant poplar deoxy‐D‐xylulose‐5‐phosphate synthase (PtDXS) by site‐directed mutagenesis improves its activity. PLoS One 11, e0161534. 27548482 10.1371/journal.pone.0161534 PMC4993486

[jam14904-bib-0008] Banerjee, A. and Sharkey, T.D. (2014) Methylerythritol 4‐phosphate (MEP) pathway metabolic regulation. Nat Prod Rep 31, 1043–1055. 24921065 10.1039/c3np70124g

[jam14904-bib-0009] Banerjee, A. , Wu, Y. , Banerjee, R. , Li, Y. , Yan, H. and Sharkey, T.D. (2013) Feedback Inhibition of deoxy‐d‐xylulose‐5‐phosphate synthase regulates the methylerythritol 4‐phosphate pathway. J Biol Chem 288, 16926–16936. 23612965 10.1074/jbc.M113.464636 PMC3675625

[jam14904-bib-0010] Barbosa, J. , Caetano, T. and Mendo, S. (2015) Class I and class II lanthipeptides produced by *Bacillus* spp. J Nat Prod 78, 2850–2866. 26448102 10.1021/np500424y

[jam14904-bib-0011] Biggs, B.W. , Lim, C.G. , Sagliani, K. , Shankar, S. , Stephanopoulos, G. , De Mey, M. and Ajikumar, P.K. (2016) Overcoming heterologous protein interdependency to optimize P450‐mediated Taxol precursor synthesis in *Escherichia coli* . Proc Natl Acad Sci 113, 3209–3214. 26951651 10.1073/pnas.1515826113 PMC4812725

[jam14904-bib-0012] Bitok, J.K. and Meyers, C.F. (2012) 2 C‐methyl‐d‐erythritol 4‐phosphate enhances and sustains cyclodiphosphate synthase IspF activity. ACS Chem Biol 7, 1702–1710. 22839733 10.1021/cb300243w PMC3477264

[jam14904-bib-0013] Bosak, T. , Losick, R.M. and Pearson, A. (2008) A polycyclic terpenoid that alleviates oxidative stress. Proc Natl Acad Sci 105, 6725–6729. 18436644 10.1073/pnas.0800199105 PMC2373358

[jam14904-bib-0014] Butcher, R.A. , Schroeder, F.C. , Fischbach, M.A. , Straight, P.D. , Kolter, R. , Walsh, C.T. and Clardy, J. (2007) The identification of bacillaene, the product of the PksX megacomplex in *Bacillus subtilis* . Proc Natl Acad Sci 104, 1506–1509. 17234808 10.1073/pnas.0610503104 PMC1785240

[jam14904-bib-0015] Cai, D. , He, P. , Lu, X. , Zhu, C. , Zhu, J. , Zhan, Y. , Wang, Q. , Wen, Z. *et al*. (2017) A novel approach to improve poly‐γ‐glutamic acid production by NADPH regeneration in *Bacillus licheniformis* WX‐02. Sci Rep 7, 43404. 28230096 10.1038/srep43404 PMC5322528

[jam14904-bib-0016] Calderone, C.T. , Kowtoniuk, W.E. , Kelleher, N.L. , Walsh, C.T. and Dorrestein, P.C. (2006) Convergence of isoprene and polyketide biosynthetic machinery: isoprenyl‐S‐carrier proteins in the pksX pathway of *Bacillus subtilis* . Proc Natl Acad Sci USA 103, 8977–8982. 16757561 10.1073/pnas.0603148103 PMC1482551

[jam14904-bib-0017] Campobasso, N. , Patel, M. , Wilding, I.E. , Kallender, H. , Rosenberg, M. and Gwynn, M.N. (2004) *Staphylococcus aureus* 3‐hydroxy‐3‐methylglutaryl‐CoA synthase. J Biol Chem 279, 44883–44888. 15292254 10.1074/jbc.M407882200

[jam14904-bib-0018] Castillo‐Hair, S. , Fujita, M. , Igoshin, O.A. and Tabor, J.J. (2019) An engineered *B. subtilis* inducible promoter system with over 10,000‐fold dynamic range. ACS Synth Biol acssynbio.8b00469. 10.1021/acssynbio.8b00469 PMC9724499 31181163

[jam14904-bib-0019] Caulier, S. , Nannan, C. , Gillis, A. , Licciardi, F. , Bragard, C. and Mahillon, J. (2019) Overview of the antimicrobial compounds produced by members of the *Bacillus subtilis* group. Front Microbiol 10, 10.3389/fmicb.2019.00302 PMC6401651 30873135

[jam14904-bib-0020] Covello, P.S. (2008) Making artemisinin. Phytochemistry 69, 2881–2885. 18977499 10.1016/j.phytochem.2008.10.001

[jam14904-bib-0021] Cui, S. , Lv, X. , Wu, Y. , Li, J. , Du, G. , Ledesma‐Amaro, R. and Liu, L. (2019) Engineering a bifunctional Phr60‐Rap60‐Spo0A quorum‐sensing molecular switch for dynamic fine‐tuning of menaquinone‐7 synthesis in *Bacillus subtilis* . ACS Synth Biol 8, 1826–1837. 31257862 10.1021/acssynbio.9b00140

[jam14904-bib-0022] Dewick, P.M. (2002) The biosynthesis of C5–C25 terpenoid compounds. Nat Prod Rep 19, 181–222. 12013278 10.1039/b002685i

[jam14904-bib-0023] Dietrich, J.A. , Yoshikuni, Y. , Fisher, K.J. , Woolard, F.X. , Ockey, D. , McPhee, D.J. , Renninger, N.S. , Chang, M.C.Y. *et al*. (2009) A novel semi‐biosynthetic route for artemisinin production using engineered substrate‐promiscuous P450 BM3. ACS Chem Biol 4, 261–267. 19271725 10.1021/cb900006h

[jam14904-bib-0024] Dong, H. and Zhang, D. (2014) Current development in genetic engineering strategies of *Bacillus* species. Microb Cell Fact 13, 63. 24885003 10.1186/1475-2859-13-63 PMC4030025

[jam14904-bib-0025] Duan, Y.X. , Chen, T. , Chen, X. and Zhao, X.M. (2010) Overexpression of glucose‐6‐phosphate dehydrogenase enhances riboflavin production in *Bacillus subtilis* . Appl Microbiol Biotechnol 85, 1907–1914. 19779711 10.1007/s00253-009-2247-6

[jam14904-bib-0026] Dueber, J.E. , Wu, G.C. , Malmirchegini, G.R. , Moon, T.S. , Petzold, C.J. , Ullal, A.V. , Prather, K.L.J. and Keasling, J.D. (2009) Synthetic protein scaffolds provide modular control over metabolic flux. Nat Biotechnol 27, 753–759. 19648908 10.1038/nbt.1557

[jam14904-bib-0027] Engels, B. , Dahm, P. and Jennewein, S. (2008) Metabolic engineering of taxadiene biosynthesis in yeast as a first step towards Taxol (Paclitaxel) production. Metab Eng 10, 201–206. 18485776 10.1016/j.ymben.2008.03.001

[jam14904-bib-0028] Fall, R. and Copley, S.D. (2000) Bacterial sources and sinks of isoprene, a reactive atmospheric hydrocarbon. Environ Microbiol 2, 123–130. 11220299 10.1046/j.1462-2920.2000.00095.x

[jam14904-bib-0029] Feng, X. , Hu, Y. , Zheng, Y. , Zhu, W. , Li, K. , Huang, C.‐H. , Ko, T.‐P. , Ren, F. *et al*. (2014) Structural and functional analysis of *Bacillus subtilis* YisP reveals a role of its product in biofilm production. Chem Biol 21, 1557–1563. 25308276 10.1016/j.chembiol.2014.08.018 PMC4252625

[jam14904-bib-0030] Ge, D. , Xue, Y. and Ma, Y. (2016) Two unexpected promiscuous activities of the iron–sulfur protein IspH in production of isoprene and isoamylene. Microb Cell Fact 15, 79. 27169371 10.1186/s12934-016-0476-9 PMC4864966

[jam14904-bib-0031] Girhard, M. , Klaus, T. , Khatri, Y. , Bernhardt, R. and Urlacher, V.B. (2010) Characterization of the versatile monooxygenase CYP109B1 from *Bacillus subtilis* . Appl Microbiol Biotechnol 87, 595–607. 20186410 10.1007/s00253-010-2472-z

[jam14904-bib-0032] Girhard, M. , Machida, K. , Itoh, M. , Schmid, R.D. , Arisawa, A. and Urlacher, V.B. (2009) Regioselective biooxidation of (+)‐valencene by recombinant *E. coli* expressing CYP109B1 from *Bacillus subtilis* in a two‐liquid‐phase system. Microb Cell Fact 8, 36. 19591681 10.1186/1475-2859-8-36 PMC2717049

[jam14904-bib-0033] Gomaa, L. , Loscar, M.E. , Zein, H.S. , Abdel‐Ghaffar, N. , Abdelhadi, A.A. , Abdelaal, A.S. and Abdallah, N.A. (2017) Boosting isoprene production via heterologous expression of the Kudzu isoprene synthase gene (kIspS) into *Bacillus* spp. cell factory. AMB Express 7, 10.1186/s13568-017-0461-7 PMC5548705 28791618

[jam14904-bib-0034] Grabowska, D. and Chelstowska, A. (2003) The ALD6 gene product is indispensable for providing NADPH in yeast cells lacking glucose‐6‐phosphate dehydrogenase activity. J Biol Chem 278, 13984–13988. 12584194 10.1074/jbc.M210076200

[jam14904-bib-0035] Gräwert, T. , Kaiser, J. , Zepeck, F. , Laupitz, R. , Hecht, S. , Amslinger, S. , Schramek, N. , Schleicher, E. *et al*. (2004) IspH protein of *Escherichia coli*: studies on iron−sulfur cluster implementation and catalysis. J Am Chem Soc 126, 12847–12855. 15469281 10.1021/ja0471727

[jam14904-bib-0036] Gu, Y. , Xu, X. , Wu, Y. , Niu, T. , Liu, Y. , Li, J. , Du, G. and Liu, L. (2018) Advances and prospects of *Bacillus subtilis* cellular factories: from rational design to industrial applications. Metab Eng 50, 109–121. 29775652 10.1016/j.ymben.2018.05.006

[jam14904-bib-0037] Guan, Z. , Xue, D. , Abdallah, I.I. , Dijkshoorn, L. , Setroikromo, R. , Lv, G. and Quax, W.J. (2015) Metabolic engineering of *Bacillus subtilis* for terpenoid production. Appl Microbiol Biotechnol 99, 9395–9406. 26373726 10.1007/s00253-015-6950-1 PMC4628092

[jam14904-bib-0038] Guiziou, S. , Sauveplane, V. , Chang, H.‐J. , Clerté, C. , Declerck, N. , Jules, M. and Bonnet, J. (2016) A part toolbox to tune genetic expression in *Bacillus subtilis* . Nucleic Acids Res gkw624. 10.1093/nar/gkw624 PMC5009755 27402159

[jam14904-bib-0039] Gustafsson, M.C.U. , Roitel, O. , Marshall, K.R. , Noble, M.A. , Chapman, S.K. , Pessegueiro, A. , Fulco, A.J. , Cheesman, M.R. *et al*. (2004) Expression, purification, and characterization of *Bacillus subtilis* cytochromes P450 CYP102A2 and CYP102A3: flavocytochrome homologues of P450 BM3 from *Bacillus megaterium* . Biochemistry 43, 5474–5487. 15122913 10.1021/bi035904m

[jam14904-bib-0040] Hahn, F.M. , Hurlburt, A.P. and Poulter, C.D. (1999) *Escherichia coli* open reading frame 696 is *idi*, a nonessential gene encoding isopentenyl diphosphate isomerase. J Bacteriol 181, 4499–4504. 10.1128/JB.181.15.4499-4504.1999 10419945 PMC103578

[jam14904-bib-0041] Hazra, A. , Chatterjee, A. and Begley, T.P. (2009) Biosynthesis of the thiamin thiazole in *Bacillus subtilis*: identification of the product of the thiazole synthase‐catalyzed reaction. J Am Chem Soc 131, 3225–3229. 19216519 10.1021/ja806752h PMC2765510

[jam14904-bib-0042] Hedl, M. , Sutherlin, A. , Wilding, E.I. , Mazzulla, M. , Mcdevitt, D. , Lane, P. , Ii, J.W.B. , Lehnbeuter, K.R. *et al*. (2002) *Enterococcus faecalis* acetoacetyl‐coenzyme A thiolase/3‐hydroxy‐3‐methylglutaryl‐coenzyme a reductase, a dual‐function protein of isopentenyl diphosphate. Biosynthesis 184, 2116–2122. 10.1128/JB.184.8.2116-2122.2002 PMC134966 11914342

[jam14904-bib-0043] Hess, B.M. , Xue, J. , Markillie, L.M. , Taylor, R.C. , Wiley, H.S. , Ahring, B.K. and Linggi, B. (2013) Coregulation of terpenoid pathway genes and prediction of isoprene production in *Bacillus subtilis* using transcriptomics. PLoS One 8, e66104. 23840410 10.1371/journal.pone.0066104 PMC3686787

[jam14904-bib-0044] Heuston, S. , Begley, M. , Davey, M.S. , Eberl, M. , Casey, P.G. , Hill, C. and Gahan, C.G.M. (2012) HmgR, a key enzyme in the mevalonate pathway for isoprenoid biosynthesis, is essential for growth of *Listeria monocytogenes* EGDe. Microbiology 158, 1684–1693. 22504435 10.1099/mic.0.056069-0

[jam14904-bib-0045] Hill, R.E. , Sayer, B.G. and Spenser, I.D. (1989) Biosynthesis of vitamin B6: incorporation of D‐1‐deoxyxylulose. J Am Chem Soc 111, 1916–1917.

[jam14904-bib-0046] Hoeffler, J.‐F. , Tritsch, D. , Grosdemange‐Billiard, C. and Rohmer, M. (2002) Isoprenoid biosynthesis via the methylerythritol phosphate pathway. Eur J Biochem 269, 4446–4457. 12230556 10.1046/j.1432-1033.2002.03150.x

[jam14904-bib-0047] Hult, K. and Berglund, P. (2007) Enzyme promiscuity: mechanism and applications. Trends Biotechnol 25, 231–238. 17379338 10.1016/j.tibtech.2007.03.002

[jam14904-bib-0048] Jin, Y. , Liu, Z. , Li, Y. , Liu, W. , Tao, Y. and Wang, G. (2016) A structural and functional study on the 2‐C‐methyl‐d‐erythritol‐4‐phosphate cytidyltransferase (IspD) from *Bacillus subtilis* . Sci Rep 6, 36379. 27821871 10.1038/srep36379 PMC5099578

[jam14904-bib-0049] Julsing, M.K. , Rijpkema, M. , Woerdenbag, H.J. , Quax, W.J. and Kayser, O. (2007) Functional analysis of genes involved in the biosynthesis of isoprene in *Bacillus subtilis* . Appl Microbiol Biotechnol 75, 1377–1384. 17458547 10.1007/s00253-007-0953-5 PMC1914294

[jam14904-bib-0050] Kalinowska‐Tłuścik, J. , Miallau, L. , Gabrielsen, M. , Leonard, G.A. , McSweeney, S.M. and Hunter, W.N. (2010) A triclinic crystal form of *Escherichia coli* 4‐diphosphocytidyl‐2 C‐methyl‐D‐erythritol kinase and reassessment of the quaternary structure. Acta Crystallogr Sect F Struct Biol Cryst Commun 66, 237–241. 10.1107/S1744309109054591 PMC2833027 20208151

[jam14904-bib-0051] Kim, J.‐E. , Jang, I.‐S. , Sung, B.H. , Kim, S.C. and Lee, J.Y. (2018) Rerouting of NADPH synthetic pathways for increased protopanaxadiol production in *Saccharomyces cerevisiae* . Sci Rep 8, 15820. 30361526 10.1038/s41598-018-34210-3 PMC6202386

[jam14904-bib-0052] Kingston, A.W. , Zhao, H. , Cook, G.M. and Helmann, J.D. (2014) Accumulation of heptaprenyl diphosphate sensitizes *Bacillus subtilis* to bacitracin: implications for the mechanism of resistance mediated by the BceAB transporter. Mol Microbiol 93, 37–49. 24806199 10.1111/mmi.12637 PMC4077933

[jam14904-bib-0053] Kontnik, R. , Bosak, T. , Butcher, R.A. , Brocks, J.J. , Losick, R. , Clardy, J. and Pearson, A. (2008) Sporulenes, heptaprenyl metabolites from *Bacillus subtilis* spores. Org Lett 10, 3551–3554. 18630919 10.1021/ol801314k PMC2646877

[jam14904-bib-0054] Kowalska, E. and Kozik, A. (2008) The genes and enzymes involved in the biosynthesis of thiamin and thiamin diphosphate in yeasts. Cell Mol Biol Lett 13, 10.2478/s11658-007-0055-5 PMC6275658 18161008

[jam14904-bib-0055] Kudoh, K. , Kubota, G. , Fujii, R. , Kawano, Y. and Ihara, M. (2017) Exploration of the 1‐deoxy‐d‐xylulose 5‐phosphate synthases suitable for the creation of a robust isoprenoid biosynthesis system. J Biosci Bioeng 123, 300–307. 27856234 10.1016/j.jbiosc.2016.10.005

[jam14904-bib-0056] Kuzma, J. , Nemecek‐Marshall, M. , Pollock, W.H. and Fall, R. (1995) Bacteria produce the volatile hydrocarbon isoprene. Curr Microbiol 30, 97–103. 7765889 10.1007/BF00294190

[jam14904-bib-0057] Kuzuyama, T. and Seto, H. (2003) Diversity of the biosynthesis of the isoprene units. Nat Prod Rep 20, 171–183. 12735695 10.1039/b109860h

[jam14904-bib-0058] Lange, B.M. , Rujan, T. , Martin, W. and Croteau, R. (2000) Isoprenoid biosynthesis: the evolution of two ancient and distinct pathways across genomes. Proc Natl Acad Sci 97, 13172–13177. 11078528 10.1073/pnas.240454797 PMC27197

[jam14904-bib-0059] Laupitz, R. , Hecht, S. , Amslinger, S. , Zepeck, F. , Kaiser, J. , Richter, G. , Schramek, N. , Steinbacher, S. *et al*. (2004) Biochemical characterization of *Bacillus subtilis* type II isopentenyl diphosphate isomerase, and phylogenetic distribution of isoprenoid biosynthesis pathways. Eur J Biochem 271, 2658–2669. 15206931 10.1111/j.1432-1033.2004.04194.x

[jam14904-bib-0060] Lawson, R.J. , Von Wachenfeldt, C. , Haq, I. , Perkins, J. and Munro, A.W. (2004) Expression and characterization of the two flavodoxin proteins of *Bacillus subtilis*, YkuN and YkuP: Biophysical properties and interactions with cytochrome P450 bioI. Biochemistry 43, 12390–12409. 15449930 10.1021/bi049131t

[jam14904-bib-0061] Lee, H. and Kim, H.Y. (2011) Lantibiotics, class I bacteriocins from the genus Bacillus. J Microbiol Biotechnol 21, 229–235. 10.4014/jmb.1010.10017. 21464591

[jam14904-bib-0062] Lee, M. , Gräwert, T. , Quitterer, F. , Rohdich, F. , Eppinger, J. , Eisenreich, W. , Bacher, A. and Groll, M. (2010) Biosynthesis of isoprenoids: crystal structure of the [4Fe‐4S] cluster protein IspG. J Mol Biol 404, 600–610. 20932974 10.1016/j.jmb.2010.09.050

[jam14904-bib-0063] Lee, W.‐H. , Kim, J.‐W. , Park, E.‐H. , Han, N.S. , Kim, M.‐D. and Seo, J.‐H. (2013a) Effects of NADH kinase on NADPH‐dependent biotransformation processes in *Escherichia coli* . Appl Microbiol Biotechnol 97, 1561–1569. 23053084 10.1007/s00253-012-4431-3

[jam14904-bib-0064] Lee, W.‐H. , Kim, M.‐D. , Jin, Y.‐S. and Seo, J.‐H. (2013b) Engineering of NADPH regenerators in *Escherichia coli* for enhanced biotransformation. Appl Microbiol Biotechnol 97, 2761–2772. 23420268 10.1007/s00253-013-4750-z

[jam14904-bib-0065] Leonard, E. , Ajikumar, P.K. , Thayer, K. , Xiao, W.‐H. , Mo, J.D. , Tidor, B. , Stephanopoulos, G. and Prather, K.L.J. (2010) Combining metabolic and protein engineering of a terpenoid biosynthetic pathway for overproduction and selectivity control. Proc Natl Acad Sci USA 107, 13654–13659. 20643967 10.1073/pnas.1006138107 PMC2922259

[jam14904-bib-0066] Li, M. , Nian, R. , Xian, M. and Zhang, H. (2018) Metabolic engineering for the production of isoprene and isopentenol by *Escherichia coli* . Appl Microbiol Biotechnol 102, 7725–7738. 30006784 10.1007/s00253-018-9200-5 PMC6132537

[jam14904-bib-0067] Liang, H. , Hu, Z. , Zhang, T. , Gong, T. , Chen, J. , Zhu, P. , Li, Y. and Yang, J. (2017) Production of a bioactive unnatural ginsenoside by metabolically engineered yeasts based on a new UDP‐glycosyltransferase from *Bacillus subtilis* . Metab Eng 44, 60–69. 28778764 10.1016/j.ymben.2017.07.008

[jam14904-bib-0068] Liu, Y.‐L. , Guerra, F. , Wang, K. , Wang, W. , Li, J. , Huang, C. , Zhu, W. , Houlihan, K. *et al*. (2012) Structure, function and inhibition of the two‐ and three‐domain 4Fe‐4S IspG proteins. Proc Natl Acad Sci 109, 8558–8563. 22586085 10.1073/pnas.1121107109 PMC3365180

[jam14904-bib-0069] Liu, Z. , Jin, Y. , Liu, W. , Tao, Y. and Wang, G. (2018) Crystal structure of IspF from *Bacillus subtilis* and absence of protein complex assembly amongst IspD/IspE/IspF enzymes in the MEP pathway. Biosci Rep 38, 10.1042/BSR20171370. PMC5821942 29335298

[jam14904-bib-0070] Lombard, J. and Moreira, D. (2011) Origins and early evolution of the mevalonate pathway of isoprenoid biosynthesis in the three domains of life. Mol Biol Evol 28, 87–99. 20651049 10.1093/molbev/msq177

[jam14904-bib-0071] Lynch, M. and Marinov, G.K. (2015) The bioenergetic costs of a gene. Proc Natl Acad Sci 112, 15690–15695. 26575626 10.1073/pnas.1514974112 PMC4697398

[jam14904-bib-0072] Ma, S.M. , Garcia, D.E. , Redding‐Johanson, A.M. , Friedland, G.D. , Chan, R. , Batth, T.S. , Haliburton, J.R. , Chivian, D. *et al*. (2011) Optimization of a heterologous mevalonate pathway through the use of variant HMG‐CoA reductases. Metab Eng 13, 588–597. 21810477 10.1016/j.ymben.2011.07.001

[jam14904-bib-0073] Ma, Y. , McClure, D.D. , Somerville, M.V. , Proschogo, N.W. , Dehghani, F. , Kavanagh, J.M. and Coleman, N.V. (2019) Metabolic engineering of the MEP pathway in *Bacillus subtilis* for increased biosynthesis of menaquinone‐7. ACS Synth Biol 8, 1620–1630. 31250633 10.1021/acssynbio.9b00077

[jam14904-bib-0074] Mahdinia, E. , Demirci, A. and Berenjian, A. (2018) Enhanced vitamin K (menaquinone‐7) production by *Bacillus subtilis* natto in biofilm reactors by optimization of glucose‐based medium. Curr Pharm Biotechnol 19, 917–924. 30474527 10.2174/1389201020666181126120401

[jam14904-bib-0075] Martin, V.J.J. , Pitera, D.J. , Withers, S.T. , Newman, J.D. and Keasling, J.D. (2003) Engineering a mevalonate pathway in *Escherichia coli* for production of terpenoids. Nat Biotechnol 21, 796–802. 12778056 10.1038/nbt833

[jam14904-bib-0076] Martin, V.J.J. , Yoshikuni, Y. and Keasling, J.D. (2001) The in vivo synthesis of plant sesquiterpenes by *Escherichia coli* . Biotechnol Bioeng 75, 497–503. 11745124 10.1002/bit.10037

[jam14904-bib-0077] Martínez, I. , Zhu, J. , Lin, H. , Bennett, G.N. and San, K.‐Y. (2008) Replacing *Escherichia coli* NAD‐dependent glyceraldehyde 3‐phosphate dehydrogenase (GAPDH) with a NADP‐dependent enzyme from *Clostridium acetobutylicum* facilitates NADPH dependent pathways. Metab Eng 10, 352–359. 18852061 10.1016/j.ymben.2008.09.001

[jam14904-bib-0078] Meadows, A.L. , Hawkins, K.M. , Tsegaye, Y. , Antipov, E. , Kim, Y. , Raetz, L. , Dahl, R.H. , Tai, A. *et al*. (2016) Rewriting yeast central carbon metabolism for industrial isoprenoid production. Nature 537, 694–697. 27654918 10.1038/nature19769

[jam14904-bib-0079] Meijer, W.J.J. , Wisman, G.B.A. , Terpstra, P. , Thorsted, P.B. , Thomas, C.M. , Holsappel, S. , Venema, G. and Bron, S. (1998) Rolling‐circle plasmids from *Bacillus subtilis*: Complete nucleolide sequences and analyses of genes of pTA1015, pTA1040, pTA1050 and pTA1060, and comparisons with related plasmids from Gram‐positive bacteria. FEMS Microbiol Rev. 10.1111/j.1574-6976.1998.tb00357.x 9532747

[jam14904-bib-0080] Moszer, I. , Rocha, E.P. and Danchin, A. (1999) Codon usage and lateral gene transfer in *Bacillus subtilis* . Curr Opin Microbiol 2, 524–528. 10508724 10.1016/s1369-5274(99)00011-9

[jam14904-bib-0081] Nguyen, H.D. , Nguyen, Q.A. , Ferreira, R.C. , Ferreira, L.C.S. , Tran, L.T. and Schumann, W. (2005) Construction of plasmid‐based expression vectors for *Bacillus subtilis* exhibiting full structural stability. Plasmid 54, 241–248. 16005967 10.1016/j.plasmid.2005.05.001

[jam14904-bib-0082] Noike, M. , Ambo, T. , Kikuchi, S. , Suzuki, T. , Yamashita, S. , Takahashi, S. , Kurokawa, H. , Mahapatra, S. *et al*. (2008) Product chain‐length determination mechanism of Z, E‐farnesyl diphosphate synthase. Biochem Biophys Res Commun 377, 17–22. 18790692 10.1016/j.bbrc.2008.09.014 PMC4747032

[jam14904-bib-0083] Patel, H. , Nemeria, N.S. , Brammer, L.A. , Freel Meyers, C.L. and Jordan, F. (2012) Observation of thiamin‐bound intermediates and microscopic rate constants for their interconversion on 1‐deoxy‐xylulose 5‐phosphate synthase: 600‐fold rate acceleration of pyruvate decarboxylation by glyceraldehyde‐3‐phosphate. J Am Chem Soc 134, 18374–18379. 23072514 10.1021/ja307315u PMC3494461

[jam14904-bib-0084] Popp, P.F. , Dotzler, M. , Radeck, J. , Bartels, J. and Mascher, T. (2017) The Bacillus BioBrick Box 2.0: expanding the genetic toolbox for the standardized work with *Bacillus subtilis* . Sci Rep 7, 15058. 29118374 10.1038/s41598-017-15107-z PMC5678133

[jam14904-bib-0085] Puan, K.J. , Wang, H. , Dairi, T. , Kuzuyama, T. and Morita, C.T. (2005) fldA is an essential gene required in the 2‐C‐methyl‐D‐erythritol 4‐phosphate pathway for isoprenoid biosynthesis. FEBS Lett 579, 3802–3806. 15978585 10.1016/j.febslet.2005.05.047

[jam14904-bib-0086] Radeck, J. , Kraft, K. , Bartels, J. , Cikovic, T. , Dürr, F. , Emenegger, J. , Kelterborn, S. , Sauer, C. *et al*. (2013) The Bacillus BioBrick Box: generation and evaluation of essential genetic building blocks for standardized work with *Bacillus subtilis* . J Biol Eng 7, 29. 24295448 10.1186/1754-1611-7-29 PMC4177231

[jam14904-bib-0087] Rahmat, E. and Kang, Y. (2020) Yeast metabolic engineering for the production of pharmaceutically important secondary metabolites. Appl Microbiol Biotechnol 104, 4659–4674. 32270249 10.1007/s00253-020-10587-y

[jam14904-bib-0088] Richard, S.B. , Bowman, M.E. , Kwiatkowski, W. , Kang, I. , Chow, C. , Lillo, A.M. , Cane, D.E. and Noel, J.P. (2001) Structure of 4‐diphosphocytidyl‐2‐C‐methylerythritol synthetase involved in mevalonate‐independent isoprenoid biosynthesis. Nat Struct Biol 8, 641–648. 11427897 10.1038/89691

[jam14904-bib-0089] Rohdich, F. , Hecht, S. , Gartner, K. , Adam, P. , Krieger, C. , Amslinger, S. , Arigoni, D. , Bacher, A. *et al*. (2002) Studies on the nonmevalonate terpene biosynthetic pathway: Metabolic role of IspH (LytB) protein. Proc Natl Acad Sci 99, 1158–1163. 11818558 10.1073/pnas.032658999 PMC122160

[jam14904-bib-0090] Schallmey, M. , Singh, A. and Ward, O.P. (2004) Developments in the use of Bacillus species for industrial production. Can J Microbiol 50, 1–17. 15052317 10.1139/w03-076

[jam14904-bib-0091] Schempp, F.M. , Drummond, L. , Buchhaupt, M. and Schrader, J. (2018) Microbial cell factories for the production of terpenoid flavor and fragrance compounds. J Agric Food Chem 66, 2247–2258. 28418659 10.1021/acs.jafc.7b00473

[jam14904-bib-0092] Seo, D. , Kamino, K. , Inoue, K. and Sakurai, H. (2004) Purification and characterization of ferredoxin‐NADP+ reductase encoded by *Bacillus subtilis* yumC. Arch Microbiol 182, 80–89. 15252706 10.1007/s00203-004-0701-5

[jam14904-bib-0093] Shields, D.C. and Sharp, P.M. (1987) Synonymous codon usage in *Bacillus subtilis* reflects both translational selection and mutational biases. Nucleic Acids Res. 10.1093/nar/15.19.8023 PMC306324 3118331

[jam14904-bib-0094] Sivy, T.L. , Fall, R. and Rosenstiel, T.N. (2011) Evidence of isoprenoid precursor toxicity in *Bacillus subtilis* . Biosci Biotechnol Biochem 75, 2376–2383. 22146731 10.1271/bbb.110572

[jam14904-bib-0095] Sivy, T.L. , Shirk, M.C. and Fall, R. (2002) Isoprene synthase activity parallels fluctuations of isoprene release during growth of *Bacillus subtilis* . Biochem Biophys Res Commun 294, 71–75. 12054742 10.1016/S0006-291X(02)00435-7

[jam14904-bib-0096] Song, Y. , Guan, Z. , van Merkerk, R. , Pramastya, H. , Abdallah, I.I. , Setroikromo, R. and Quax, W.J. (2020) Production of squalene in *Bacillus subtilis* by squalene synthase screening and metabolic engineering. J Agric Food Chem 68, 4447–4455. 32208656 10.1021/acs.jafc.0c00375 PMC7168599

[jam14904-bib-0097] Span, I. , Wang, K. , Wang, W. , Zhang, Y. , Bacher, A. , Eisenreich, W. , Li, K. , Schulz, C. *et al*. (2012) Discovery of acetylene hydratase activity of the iron‐sulphur protein IspH. Nat Commun 3, 10.1038/ncomms2052. PMC3745992 22948824

[jam14904-bib-0098] Steinbacher, S. , Kaiser, J. , Gerhardt, S. , Eisenreich, W. , Huber, R. , Bacher, A. and Rohdich, F. (2003) Crystal structure of the type II isopentenyl diphosphate: dimethylallyl diphosphate isomerase from*Bacillus subtili*s. J Mol Biol 329, 973–982. 12798687 10.1016/s0022-2836(03)00527-8

[jam14904-bib-0099] Toymentseva, A.A. and Altenbuchner, J. (2019) New CRISPR‐Cas9 vectors for genetic modifications of *Bacillus* species. FEMS Microbiol Lett 366. 10.1093/femsle/fny284 30520985

[jam14904-bib-0100] Tsuruta, H. , Paddon, C.J. , Eng, D. , Lenihan, J.R. , Horning, T. , Anthony, L.C. , Regentin, R. , Keasling, J.D. *et al*. (2009) High‐level production of amorpha‐4, 11‐diene, a precursor of the antimalarial agent artemisinin, in *Escherichia coli* . PLoS One 4, e4489. 19221601 10.1371/journal.pone.0004489 PMC2637983

[jam14904-bib-0101] Vavrová, Ľ. , Muchová, K. and Barák, I. (2010) Comparison of different *Bacillus subtilis* expression systems. Res Microbiol 161, 791–797. 20863884 10.1016/j.resmic.2010.09.004

[jam14904-bib-0102] Volke, D.C. , Rohwer, J. , Fischer, R. and Jennewein, S. (2019) Investigation of the methylerythritol 4‐phosphate pathway for microbial terpenoid production through metabolic control analysis. Microb Cell Fact 18, 192. 31690314 10.1186/s12934-019-1235-5 PMC6833178

[jam14904-bib-0103] Wagner, W.P. , Helmig, D. and Fall, R. (2000) Isoprene biosynthesis in *Bacillus subtilis* via the methylerythritol phosphate pathway. J Nat Prod 63, 37–40. 10650075 10.1021/np990286p

[jam14904-bib-0104] Wang, Y. , San, K.‐Y. and Bennett, G.N. (2013) Improvement of NADPH bioavailability in *Escherichia coli* by replacing NAD(+)‐dependent glyceraldehyde‐3‐phosphate dehydrogenase GapA with NADP (+)‐dependent GapB from *Bacillus subtilis* and addition of NAD kinase. J Ind Microbiol Biotechnol 40, 1449–1460. 24048943 10.1007/s10295-013-1335-x

[jam14904-bib-0105] Wang, Y. , Weng, J. , Waseem, R. , Yin, X. , Zhang, R. and Shen, Q. (2012) *Bacillus subtilis* genome editing using ssDNA with short homology regions. Nucleic Acids Res 40, e91. 22422839 10.1093/nar/gks248 PMC3384351

[jam14904-bib-0106] Wang, Z. , Chen, T. , Ma, X. , Shen, Z. and Zhao, X. (2011) Enhancement of riboflavin production with *Bacillus subtilis* by expression and site‐directed mutagenesis of *zwf* and *gnd* gene from *Corynebacterium glutamicum* . Bioresour Technol 102, 3934–3940. 21194928 10.1016/j.biortech.2010.11.120

[jam14904-bib-0107] Whitehouse, C.J.C. , Bell, S.G. and Wong, L.‐L. (2012) P450 BM3 (CYP102A1): connecting the dots. Chem Soc Rev 41, 1218–1260. 22008827 10.1039/c1cs15192d

[jam14904-bib-0108] Wilding, E.I. , Brown, J.R. , Bryant, A.P. , Chalker, A.F. , Holmes, D.J. , Ingraham, K.A. , Iordanescu, S. , So, C.Y. *et al*. (2000) Identification, evolution, and essentiality of the mevalonate pathway for isopentenyl diphosphate biosynthesis in gram‐positive cocci. J Bacteriol 182, 4319–4327. 10894743 10.1128/jb.182.15.4319-4327.2000 PMC101949

[jam14904-bib-0109] Withers, S.T. , Gottlieb, S.S. , Lieu, B. , Newman, J.D. and Keasling, J.D. (2007) Identification of isopentenol biosynthetic genes from *Bacillus subtilis* by a screening method based on isoprenoid precursor toxicity. Appl Environ Microbiol 73, 6277–6283. 17693564 10.1128/AEM.00861-07 PMC2075014

[jam14904-bib-0110] Wolff, M. , Seemann, M. , Bui, B. , Frapart, Y. , Tritsch, D. , Estrabot, A.G. , Rodríguez‐Concepción, M. (2003) Isoprenoid biosynthesis via the methylerythritol phosphate pathway: the ( E )‐4‐hydroxy‐3‐methylbut‐2‐enyl diphosphate reductase (LytB/IspH) from *Escherichia coli* is a [4Fe‐4S] protein. FEBS Lett 541, 115–120. 12706830 10.1016/s0014-5793(03)00317-x

[jam14904-bib-0111] Xiang, S. , Usunow, G. , Lange, G. , Busch, M. and Tong, L. (2007) Crystal structure of 1‐deoxy‐D‐xylulose 5‐phosphate synthase, a crucial enzyme for isoprenoids biosynthesis. J Biol Chem. 10.1074/jbc.M610235200 17135236

[jam14904-bib-0112] Xue, D. , Abdallah, I.I. , de Haan, I.E.M. , Sibbald, M.J.J.B. and Quax, W.J. (2015) Enhanced C30carotenoid production in *Bacillus subtilis* by systematic overexpression of MEP pathway genes. Appl Microbiol Biotechnol 99, 5907–5915. 25851715 10.1007/s00253-015-6531-3 PMC4480331

[jam14904-bib-0113] Xue, J. and Ahring, B.K. (2011) Enhancing isoprene production by genetic modification of the 1‐deoxy‐D‐Xylulose‐5‐phosphate pathway in *Bacillus subtilis* . Appl Environ Microbiol 77, 2399–2405. 21296950 10.1128/AEM.02341-10 PMC3067423

[jam14904-bib-0114] Yang, D. , Park, S.Y. , Park, Y.S. , Eun, H. and Lee, S.Y. (2020) Metabolic engineering of *Escherichia coli* for natural product biosynthesis. Trends Biotechnol 38, 745–765. 31924345 10.1016/j.tibtech.2019.11.007

[jam14904-bib-0115] Yang, S. , Cao, Y. , Sun, L. , Li, C. , Lin, X. , Cai, Z. , Zhang, G. and Song, H. (2019) Modular pathway engineering of *Bacillus subtilis* to promote de novo biosynthesis of menaquinone‐7. ACS Synth Biol 8, 70–81. 30543412 10.1021/acssynbio.8b00258

[jam14904-bib-0116] Yang, S. , Kang, Z. , Cao, W. , Du, G. and Chen, J. (2016) Construction of a novel, stable, food‐grade expression system by engineering the endogenous toxin‐antitoxin system in *Bacillus subtilis* . J Biotechnol 219, 40–47. 26721182 10.1016/j.jbiotec.2015.12.029

[jam14904-bib-0117] Yoshida, K. , Ueda, S. and Maeda, I. (2009) Carotenoid production in *Bacillus subtilis* achieved by metabolic engineering. Biotechnol Lett 31, 1789–1793. 19618272 10.1007/s10529-009-0082-6

[jam14904-bib-0118] Zhao, H. , Sun, Y. , Peters, J.M. , Gross, C.A. , Garner, E.C. and Helmann, J.D. (2016) Depletion of undecaprenyl pyrophosphate phosphatases disrupts cell envelope biogenesis in *Bacillus subtilis* . J Bacteriol 198, 2925–2935. 27528508 10.1128/JB.00507-16 PMC5055597

[jam14904-bib-0119] Zhao, J. , Li, Q. , Sun, T. , Zhu, X. , Xu, H. , Tang, J. , Zhang, X. and Ma, Y. (2013) Engineering central metabolic modules of *Escherichia coli* for improving β‐carotene production. Metab Eng 17, 42–50. 23500001 10.1016/j.ymben.2013.02.002

[jam14904-bib-0120] Zhao, X. , Shi, F. and Zhan, W. (2015) Overexpression of ZWF1 and POS5 improves carotenoid biosynthesis in recombinant *Saccharomyces cerevisiae* . Lett Appl Microbiol 61, 354–360. 26179622 10.1111/lam.12463

[jam14904-bib-0121] Zhao, Y. , Yang, J. , Qin, B. , Li, Y. , Sun, Y. , Su, S. and Xian, M. (2011) Biosynthesis of isoprene in *Escherichia coli* via methylerythritol phosphate (MEP) pathway. Appl Microbiol Biotechnol 90, 1915–1922. 21468716 10.1007/s00253-011-3199-1

[jam14904-bib-0122] Zhou, K. , Zou, R. , Zhang, C. , Stephanopoulos, G. and Too, H.‐P. (2013) Optimization of amorphadiene synthesis in *Bacillus subtilis* via transcriptional, translational, and media modulation. Biotechnol Bioeng 110, 2556–2561. 23483530 10.1002/bit.24900

[jam14904-bib-0123] Zhu, S. , Cai, D. , Liu, Z. , Zhang, B. , Li, J. , Chen, S. and Ma, X. (2019) Enhancement of bacitracin production by NADPH generation via overexpressing glucose‐6‐phosphate dehydrogenase Zwf in *Bacillus licheniformis* . Appl Biochem Biotechnol 187, 1502–1514. 30267286 10.1007/s12010-018-2894-0

